# Geographies of the COVID-19 pandemic

**DOI:** 10.1177/2043820620936050

**Published:** 2020-06-24

**Authors:** Reuben Rose-Redwood, Rob Kitchin, Elia Apostolopoulou, Lauren Rickards, Tyler Blackman, Jeremy Crampton, Ugo Rossi, Michelle Buckley

**Affiliations:** University of Victoria, Canada; Maynooth University, Ireland; University of Cambridge, UK; RMIT University, Australia; University of Victoria, Canada; Newcastle University, UK; Gran Sasso Science Institute, Italy; University of Toronto, Canada

**Keywords:** coronavirus, COVID-19, crisis, disease, inequality, pandemic, public health, SARS-CoV-2

## Abstract

The spread of the novel coronavirus (SARS-CoV-2) has resulted in the most devastating global public health crisis in over a century. At present, over 7 million people from around the world have contracted the Coronavirus Disease 2019 (COVID-19), leading to more than 400,000 deaths globally. The global health crisis unleashed by the COVID-19 pandemic has been compounded by political, economic, and social crises that have exacerbated existing inequalities and disproportionately affected the most vulnerable segments of society. The global pandemic has had profoundly geographical consequences, and as the current crisis continues to unfold, there is a pressing need for geographers and other scholars to critically examine its fallout. This introductory article provides an overview of the current special issue on the geographies of the COVID-19 pandemic, which includes 42 commentaries written by contributors from across the globe. Collectively, the contributions in this special issue highlight the diverse theoretical perspectives, methodological approaches, and thematic foci that geographical scholarship can offer to better understand the uneven geographies of the Coronavirus/COVID-19.

## Introduction

The world is again in crisis. As the coronavirus (SARS-CoV-2) and its accompanying disease (COVID-19) have spread globally since December 2019, hundreds of thousands have died, millions more have been infected with the virus, and entire economies have come to a screeching halt amid government-imposed lockdowns. The COVID-19 pandemic is, first and foremost, a global public health crisis, yet its impacts extend far beyond the realm of epidemiology alone. We are also witnessing a political, economic, and social crisis the likes of which the world has not seen since the 1918 influenza pandemic and the Great Depression (Cohan, 2020; Colvin and McLaughlin, 2020).

Border closures have restricted international travel (Salcedo et al., 2020); quarantine and stay-at-home orders have emptied city streets, parks, and public spaces (Morton, 2020); unemployment rates have skyrocketed (ILO, 2020); schools and universities have shuttered their doors as many rapidly transitioned to online courses (Lee, 2020); and nursing homes, prisons, migrant detention centers, and slaughterhouses have become hotbeds of death and disease (Mosk et al., 2020)—all of which has exacerbated existing social inequalities and economic disparities.

At the same time, there has been a tremendous outpouring of mutual aid and social solidarity to assist those in immediate need (Solnit, 2020; Tolentino, 2020). Health care and other ‘essential’ workers are putting their lives at risk on a daily basis providing medical care, keeping grocery store shelves stocked, and ensuring that public transportation systems are running (Sainato, 2020); medical researchers are racing to find a coronavirus vaccine and other medical treatments for COVID-19 (Sanger et al., 2020); labor rights advocates and trade unions are fighting to combat exploitative and unsafe working conditions (Greenhouse, 2020); and grassroots organizations are raising funds and organizing solidarity networks to support socially just responses to the global pandemic (Diavolo, 2020). Despite these efforts, the worst days of the COVID-19 pandemic may yet be to come, and much that will gain clarity with hindsight in the future remains obscured in the immediacy of the present crisis. However, one thing that is certain is that we do not have the luxury of waiting until the crisis is over before critically examining its fallout.

Although the precise origins of the SARS-CoV-2 virus are currently under investigation (*Science*, 2020), it is well-established that global pandemics are not simply ‘natural’ disasters. Rather, they are directly linked to the emergence of new pathogens in the wake of industrialized agriculture, livestock production, deforestation, and capital’s relentless exploitation of nature (Harvey, 2020; Moseley, 2020; Wallace, 2020). In the case of SARS-CoV-2, the first documented symptoms of a novel coronavirus were recorded on December 8, 2019 in Wuhan, China (WHO, 2020a). By December 31, a cluster of 41 cases had been identified and linked to a seafood market which was closed on January 1, 2020 (WHO, 2020a). The Chinese authorities isolated the new virus on January 7, later named SARS-CoV-2 (Severe Acute Respiratory Syndrome Coronavirus 2), and confirmed that it spread through human-to-human contact with infection resulting in the Coronavirus Disease 2019 (COVID-19). While the majority of cases produced mild flu-like symptoms, it was clear the disease could progress to acute respiratory distress, multi-organ failure, and death. By January 22, there were 571 confirmed cases and 17 deaths in China, with confirmed cases reported in Hong Kong, Japan, South Korea, Taiwan, and the United States (Kuo, 2020). On January 23, China imposed lockdown measures on the 57 million people living in Hubei province in an effort to limit further spread (BBC, 2020a). The first case of COVID-19 in Europe was initially recorded on January 24, although subsequent evidence suggests it had already spread to at least one European country (France) by the end of December 2019 (BBC, 2020b). The World Health Organization (WHO) declared a global health emergency on January 30 and a global pandemic on March 11. By early-to-mid March, dozens of countries had moved from a containment phase of response designed to prevent the virus from spreading (using measures such as increased hygiene as well as testing, tracing, and isolating) to a delay phase designed to reduce the peak of impact and limit overwhelming health care systems (using containment measures plus physical distancing, self-isolation, and quarantining; limiting travel and social gatherings; closing businesses; and enforcing lockdowns). As of mid-June 2020, there were over 7 million confirmed cases and more than 400,000 deaths globally, spread across at least 215 countries and territories (CDC, 2020; WHO, 2020b).

Within a relatively short period of time, the COVID-19 pandemic has become a truly global event with consequences that span every facet of daily life in ways that are profoundly geographical. The spread of the disease demanded a spatialized response that recognized the vectors and clustering of diffusion within localized settings as well as across space and borders. Spatial modeling has been an essential part of the epidemiological toolkit guiding public health and government policy responses, and maps and charts that compare places have become key media for enhancing public understanding of the pandemic. Measures put in place to contain, delay, and mitigate the diffusion of the disease have radically disrupted society and economy, transforming socio-spatial relations and socionatures, delimiting mobility and access, reconfiguring the production of space and territory, and altering perceptions of place. In turn, these changes have profoundly transformed the familial spaces of home, modes of living, and the geographies of everyday lives; relations to public space and nature; the operations of work and the space economy; the geopolitical landscape; and the global dynamics of capital accumulation.

The measures adopted in response to the COVID-19 pandemic and their effects have been far from universal—varying between and within countries depending on political regime and capacity to respond—which has led to differences in approaches taken as well as differential infection rates and deaths (Gibney, 2020). Governmental responses to the pandemic have rapidly exposed the impacts of prolonged austerity that had left public health care systems under-prepared to deal with a pandemic, particularly in the countries most severely affected by the 2008 global economic crisis. The complete absence or further dismantling of the welfare state due to the neoliberal restructuring of the last few decades has disproportionately affected people who have experienced the most hardship from increasing inequalities with impacts being reported along lines of class, race, ethnicity, and gender (Platt and Warwick, 2020).

Geography as a discipline and practice has also been transformed. University campuses have closed, academics are working from home, teaching has moved online, in-situ fieldwork has halted or shifted to the use of virtual methods where possible, and seminars, workshops, and conferences have been canceled or converted into virtual events. There has been enormous pressure to deliver existing classes and commitments, and support students and colleagues, in new ways, while also coping with changing conditions (such as working from home while providing childcare and home schooling). The pandemic and its consequent economic impacts are highlighting and exacerbating underlying crises and chronic problems in higher education in many countries across the globe. Even those universities with large endowments have been breathtakingly swift to cut academic staffing costs. Not surprisingly, the present crisis is affecting university employees unequally—with precarious faculty and staff in many universities experiencing the most hardship as their pay and work conditions deteriorate significantly and some lose their jobs altogether. A whole generation of students and scholars is facing limited future career prospects. Moreover, many of our research partners are also struggling, including collaborators in the arts sector, non-governmental organizations, and community services.

To avoid placing additional pressure on our academic peers during the initial stage of the pandemic, the editorial team at *Dialogues in Human Geography* decided to temporarily pause the refereeing of new papers for 6 weeks and granted automatic extensions for those who had already agreed to peer review manuscripts currently within the system. At the same time, there has been a desire by many to respond intellectually and constructively to the current global crisis. This has demanded a quick response to an unfolding set of interrelated events, in contrast to the often slower and more reflexive scholarship that is more typical in the academy. Indeed, geographers and other scholars are already producing intellectual and empirical analyses of COVID-19. Some are actively working with government, policy-makers, and communities, undertaking epidemiological modeling, helping with data infrastructures, advising on interventions and recovery strategies, and volunteering to help the vulnerable. Many geographers are also activists and key members of solidarity networks and grassroots groups that have been formulated to address already existing inequalities that COVID-19 has exacerbated as well as new inequalities caused by the pandemic.

Some scholars have expressed concerns that fast, not-fully-considered work may constitute academic disaster voyeurism; weak, underdeveloped scholarship; neoliberal opportunism through rapid publication; or reproduces and deepens gendered, classed, and other inequalities within the academy given the differential ability to contribute. We share those concerns and believe they deserve consideration (indeed, they are discussed by contributors in this special issue). At the same time, given the scope, extent, and impact of the current crisis, there is undoubtedly a need to respond in the here-and-now, drawing on the deep well of theoretical and applied expertise across the full breadth of the discipline. For geographers not to respond, or to wait until the crisis has passed before examining its processes and impacts, would be to forgo practicing public and engaged geography of the here-and-now. It would deny our ability, and arguably abdicate our responsibility, if we did not use our skills in geographical scholarship to help bear witness and make sense of what is happening and to help cultivate new critical publics. Moreover, it would leave interventions in the contemporary world to the preserve of politicians, journalists, civil servants, and academics in other fields. It would also consign geography to only being historical rather than analyzing or being applied in the present, which, in an increasingly disrupted world, is an untenable stance (although historical methods themselves remain crucial to understanding the present). So however uncomfortable or disastrous our present is, or whether we are all able to contribute equally, we believe it is important for geographers who feel they have something to offer to respond as best they can within their own circumstances.

As the commentaries in this special issue of *Dialogues in Human Geography*—together with recent editorials and articles in other geography journals (Castree et al., 2020; Desjardins et al., 2020; Malanson, 2020; Shi and Liu, 2020; Sparke and Anguelov, 2020)—demonstrate, the commitment to producing timely, publicly-engaged, and socially relevant scholarship is a view shared by many in the discipline. Our aim in assembling this special issue has been to curate a set of interventions that provides some initial meaningful geographical analysis of the COVID-19 pandemic and contributes to wider academic, political, and public policy debates while also mapping out future research agendas and everyday geographical praxes.

After posting a call for submissions on social media, we received 64 proposals to contribute to this special issue and have ultimately included 42 commentaries in the present issue. In making this selection, we sought to balance for thematic focus, geographic location, gender, and career stage. We were especially mindful of the gender imbalances in article submissions reported by other journals during the pandemic (Flaherty, 2020), and over 50 percent of the commentaries in this special issue have at least one female author. The commentaries also cover a wide range of issues and perspectives—including contributions by scholars from 16 different countries around the world—all of which bear witness to and aid our understanding of the unfolding crisis.

## Geographies of the Coronavirus/COVID-19

A significant theme spanning across the commentaries in this special issue involves the crucial concerns of care, reciprocity, and social reproduction, with many contributors valuably documenting and analyzing the uneven effects of the COVID-19 crisis across social classes and imagining alternative future socio-spatial relations. **Avril Maddern** makes clear what is ultimately at stake in the crisis, examining the new global geographies of death and bereavement caused by COVID-19 as well as considering other forms of loss such as unemployment and social isolation. She argues that it is crucial to map out these emotional-affective topographies, but also to highlight consolation and hope as well as creating the conditions for personal and social resilience. Mutual aid and the need for cooperation, compassion, care, and reciprocity in the differential unfolding and consequences of the pandemic are explored by **Simon Springer**, who argues that the crisis exposes the failings of capitalism and neoliberal states, and offers the prospect to reconfigure society based on an ethics of people and nature before profit. Similarly, **James Tyner and Stian Rice** argue that the crisis provides an opportunity to move away from a society organized around the circulation and accumulation of capital to one centered on living a ‘meaningful life’, and ask what geographical scholarship promoting such a shift might look like.

There is an emerging consensus that low-income communities, the working class, women, people of color, and Indigenous people are being more significantly impacted by the pandemic in relation to health, working arrangements, and social and economic hardship. Italy was the initial epicenter of the pandemic in Europe and was the first European country to implement lockdown measures. These measures had a profound impact on the gendered geographies of work and home. Based on interviews with 20 working mothers, and informal chats with 30 others, **Lidia Manzo and Alessandra Minello** discuss the unequal domestic and parenting arrangements of those working remotely and the resources they have used to create networks of social support. Similarly, **Chiara Lacovone, Alberto Valz Gris, Astrid Safina, Andrea Pollio, and Francesca Governa** reflect on their personal experiences to discuss the difficulties of pivoting to home working, practicing geographical scholarship on the margins of Anglo-American hegemony, and the emergence of new geographies of care and collegiality. **Kath Browne, Niharika Banerjea, and Leela Bakshi** discuss notions of precarity, survival, and liveability for queer women in the non-normative situation created by the pandemic and bearing witness to the grounded local realities of social life and the sustenance of transnational connections. Likewise, **LaToya Eaves and Karen Falconer Al-Hindi** argue that it is important that an intersectional feminist approach is adopted to examine and respond to uneven and unequal pandemic geographies.

How the pandemic is framed and tackled has varied across countries. It is clear that states led by right-wing populist administrations have approached public health measures and societal response in a more cavalier fashion and have used dissimulation, fake news, and blame-shifting to deny and decry outcomes. **Matheus Hoffmann Pfrimer and Ricardo Barbosa, Jr.** examine the securitized discursive strategies employed by Brazilian president Jair Bolsonaro and his administration to bolster their political message and create internal stability, while mis-managing the public health response and escalating its effects. Similarly, **Aoife Delaney** charts the scalar politics of response in the US, where a populist president and deep political divisions across states have led to a conflictual, disorganized, and variable approach as well as a large number of cases and deaths. She compares the US situation to the coordinated management and emergency response in Ireland where there has been a high degree of agreement and compliance with public health measures. Nonetheless, Ireland had been pursuing a path of austere neoliberalism which—adopting the health language of the pandemic that **Adam Standring and Jonathan Davies** describe as a ‘pre-existing condition’—exacerbates inequality and precarity resulting from counter-measures. They suggest that state interventions in such a situation may act as ‘necro-socialism’ supporting the status quo and devoid of emancipatory goals, and speculate as to alternative political framings. **Sung-Yueh Perng** documents the orderly response to COVID-19 in Taiwan, where previous encounters with coronaviruses have forged a well-coordinated set of practices that includes civic action, though there are fissures with respect to certain populations. He argues that it is unrealistic to think that there will be a return to ‘normality’, but instead we need to learn to live with and manage our co-existence with viruses and plan accordingly.

Although the COVID-19 pandemic is a global crisis, **Fenglong Wang, Sainan Zou, and Yungang Liu** argue that most responses to the pandemic have been framed around the ‘territorial trap’ of viewing the state as a fixed container of society. They consider three expressions of the territorial trap during the coronavirus pandemic as it relates to the governance of international travel and migration, inter-state coordination, and territorial thinking. One key action adopted by states has been the use of bordering practices to limit mobility, how COVID-19 spreads, and mitigate its effects. With respect to Hong Kong and China, **Xiaofeng Liu and Mia Bennett** document the securitization of medical products within territories, new biopolitics to reterritorialize communities through the use of geofencing to track and limit mobility, and the creation of new gated communities. **Kelsey Leonard** also highlights bordering practices, focusing on the disproportionate impact of COVID-19 on Indigenous nations in North America and how settler colonialism expressed through non-essential second-home escapism of urban elites has led to Indigenous responses such as checkpoints that render Indigenous borders into sites of compassionate community care. As with any new regulatory interventions, people will find ways to circumvent and subvert them. **Heidi Østbø Haugen and Angela Lehmann** discuss how the border controls put in place to limit movement from China to Australia were bypassed by international students staying in a third country en route, thus externalizing the risk of infection and maintaining the profitable international student market. **Benjamin Lucca Iaquinto** details the role that tourism played as a vector in the diffusion of the disease and argues that studies of mobility politics have much to contribute to the study of COVID-19. Taking a different tack, **Miriam Tedeschi** explores the spatialities and materialities of bordering practices in the pandemic from a geographic and legal perspective through an autoethnography of traveling from Italy to Finland as a potentially contaminated body while new response measures were being put in place. How tourists and those moving between nations, especially travelers from East Asia, have been racialized, demonized, and geopolitically recast in an international blame-game during the pandemic is examined by **Mary Mostafanezhad, Joseph Cheer, and Harng Luh Sin**. They argue that there are signs that future travel will be mediated by new regulatory arrangements designed to reassure and manage geopolitical anxieties. **Autumn James** maps such anxieties and perceptions of risk onto more local geographies, exploring how assessments of safety, and fears of the virus or dismissal of its effects, influence spatial behavior and decision-making.

Lockdown and other measures have had a dramatic impact on the economy at all scales from local labor markets to global production networks (GPN). The interruption and slow-down in economic activity has, **Callum Ward** argues, led to a rupture in the circulation of fictitious capital and the temporal logics of financialized capitalism that will reverberate for some time, resulting in the annihilation of time by space. This is evident in the fashion industry, as **Taylor Brydges and Mary Hanlon** explain, where spatio-temporal disruptions to economic production and consumption have created a crisis that further entrenches existing structural inequalities and disproportionately affects labor across GPNs, particularly among garment workers. **Sabina Lawreniuk** focuses her analysis specifically on the plight of garment workers in Cambodia and the consequences of unemployment on daily life, arguing that their hyper-precarity quickly made them the victims of necrocapitalist logics of GPNs. Similarly, many gig workers have seen a significant contraction in work and income. However, while many are experiencing hardship, **Srujana Katta, Adam Badger, Mark Graham, Kelle Howson, Funda Ustek-Spilda, and Alessio Bertolini** argue that some platform companies, such as Uber, have, under pressure, moved away from a disembedded position that avoids local norms and regulations and supports precarity. Instead, they are adopting a more embedded stance through the provision of modest employment benefits signaling a possible shift in labor relations post-crisis. The opportunity and ability for workers to pivot to working from home, as **Darja Reuschke and Alan Felstead** explore, has reinforced marked social and spatial inequalities between those able to make the transition and those who have been furloughed or lost jobs. Beyond the reconfiguration of work practices and implications for the future workplace landscape, they argue that places with concentrations of sectors amenable to working from home are likely to be more resilient and quicker to recover.

**Creighton Connolly, Harris Ali, and Roger Keil** highlight urban life and urbanization as key factors giving rise to the pandemic through shifting urban-ecological dynamics, dense networks, interdependent infrastructure, and inter-urban connectivity, while also being key sites for mitigating measures. Drawing on examples from Sub-Saharan Africa, **Brandon Finn and Lindsay Kobayashi** argue that understanding how public health interventions produce durable autocratic urban governance and reproduce structural inequalities between social classes requires a historical understanding of similar deployments in past pandemics. Taking a different tack, **Michele Acuto** contends that geographers can make better sense of the urban realm in the crisis by conjoining planetary urbanism with world political systems and taking a fuller account of the dynamics of global urban governance. Focusing specifically on housing, and using Australia as an example, **Sophia Maalsen, Dallas Rogers, and Leo Patterson Ross** note that the pandemic has rendered visible long-term, systemic issues and created a new suite of problems that need redress. **Ulises Moreno-Tabarez** makes clear that the pandemic is not simply an urban phenomena, and again reiterates the need for historical contextualization, unpacking how the legacies of slavery and colonialism continue to haunt Afro-Indigenous ruralities in the Costa Chica region of Guerrero, Mexico, during the crisis.

As the COVID-19 crisis has unfolded, there has been a turn to digital technologies to help implement response measures and mitigate the effects of these measures. **Ayona Datta** calls attention to the ways in which smart technologies have been repurposed in India to track and manage the pandemic. She notes that 45 out of 100 smart cities in India have renamed their Integrated Command and Control Centres (ICCC) as ‘COVID-19 war rooms’ and a number of contact tracing and quarantine apps have been deployed, including ones that use selfies and facial recognition to monitor movement. Likewise, **Bei Chen, Simon Marvin, and Aidan While** document the accelerated development of urban robotics, autonomous systems, and artificial intelligence for urban epidemic control in China, and warn of the potential to extend state surveillance and control. For digital platform companies, the repurposing and extension of their technologies and embedding into the monitoring and lifting of restrictions is, as **Jonathan Cinnamon** contends through his analysis of Google Maps data, a key means to strengthen their economic and political position, offsetting declining advertising revenues and defraying regulation. Similar themes are explored by **Ryan Burns** who examines the transition to online education, arguing that rather than challenging the neoliberalization of education, such a move risks further deepening its reforms and embedding a pernicious techno-utopian imaginary and solutionism within the sector.

Data-driven technologies and the use of charts, dashboards, maps, and models have become a key means by which spatialized knowledge about COVID-19 is understood and circulated within expert groups and the wider public as well as informing decision-making about counter-measures and when they should be applied. **Chris Brunsdon**, a member of the COVID-19 modeling team providing analysis to the National Public Health Emergency Team that is guiding the Irish government response to the pandemic crisis, discusses technical issues with epidemiological modeling, while recognizing the need for a perspective informed by critical data studies. The data informing these models has been presented to the public using interactive dashboards. **Philip James, Ronnie Das, Agata Jalosinska, and Luke Smith** describe their work repurposing the Urban Observatory for Newcastle, UK, to provide real-time insights into the impacts of lockdown policy on urban governance. The use of dashboards is critiqued by **Jonathan Everts** who argues that they present constrained and tailored views of the unfolding pandemic and obscure localized variances and socio-spatial inequalities, potentially widening them. Similarly, **Peter Mooney and Levente Juhász** question the design and efficacy of web-based maps and raise concerns about the extent to which they have been co-opted into the ‘infodemic’ of misleading and politicized data analytics about the pandemic. **Jeremy Brice** uses the common ‘flatten the curve’ chart and the popular *Financial Times* graph comparing death rates across countries to argue that data representations actively produce possible pandemic futures through biopolitical and geopolitical registers and render other data as counterfactual. Drawing on an analysis of Twitter data concerning the unsubstantiated rumor that COVID-19 is a Chinese bioweapon that escaped from a lab in Wuhan, **Monica Stephens** explores how misinformation and conspiracy theories are established, circulate, and grow, forming part of the infodemic enabled by social media and 24/7 news cycles. Relatedly, **Kaya Barry** explores how representations and diagrammatic instructions of COVID-19 are informing and shaping how individuals enact specific spatial, mobile, and bodily practices and can accentuate public feelings of uncertainty, emergency, or threat.

The final two commentaries in this special issue concern the relationship between the pandemic and nature. **Gwendolyn Blue and Melanie Rock** explain that genomic science has become the dominant approach to understanding zoonotic disease (i.e. infectious pathogens capable of crossing the species barrier) and its transmission. They contend that this de-emphasizes other knowledges and local context, and argue for the inclusion of more-than-human accounts within discussions of health and disease. One trope of the crisis is that the pause in economic activity and human mobility has led to a resurgence of nature. Similarly advocating for a more-than-human approach, **Adam Searle and Jonathon Turnbull** argue that this trope arises from biocultural decontextualization that assumes nature has an inherent capacity to heal itself. This downplays the need for urgent environmental action obscuring that resurgence is a multispecies endeavor that requires cultivation, nurture, and care to foster conviviality.

## Conclusion

The commentaries in this special issue present a sample of the wide spectrum of approaches and perspectives that geographical scholarship is already bringing to bear on understanding the nature and workings of the unfolding COVID-19 pandemic crisis. They highlight that the COVID-19 pandemic is thoroughly spatial in nature as well as the value of geographical theory and praxis in providing critical (what is happening) and normative (what should happen) thinking as well as applied outcomes (making things happen). While much attention is being focused on scientific and technological responses to COVID-19, it is clear that the myriad of political, economic, and social crises that have accompanied the public health crisis of the pandemic require critical and reflexive analysis and theoretical insight. Such scholarship and praxis can expose the socio-spatial processes at play and their consequences, and can translate these to shape public discourse and public policy which have the potential to transform everyday lives.

The COVID-19 pandemic has created a global crisis in which the ‘normal’ conditions and structures of societies have been upended. Much of the rhetoric in 'telling the story of the pandemic' (Mathur, 2020) is about returning to ‘normality’, but it is clear that whatever happens we will be entering a ‘new normal’, whether that be altered social practices, truncated mobility, reconfigured labor relations, increased precarity, deepened inequalities, or more cooperative, communal, caring arrangements. There are also increasing concerns that governmental attempts to deal with the pandemic without further disrupting the market may ultimately lead to the emergence of a new neoliberal authoritarianism, already expressed in cities across the globe in measures that have allowed the re-opening of shopping malls and tourism but criminalize people’s gatherings in public spaces—as we have witnessed with police crackdowns on anti-racism demonstrations in response to the police killing of George Floyd. As Arundhati Roy (2020) has written, the pandemic can act as ‘a portal, a gateway between one world and the next. We can choose to walk through it, dragging the carcasses of our prejudice and hatred, our avarice, our data banks and dead ideas, our dead rivers and smoky skies behind us. Or we can walk through lightly, with little luggage, ready to imagine another world. And ready to fight for it’. In addition to documenting and explaining the socio-spatial processes driving the transformations occurring as a result of the COVID-19 pandemic, geographers need to envisage new geographical imaginations and help fight to realize them. The commentaries in this special issue provide starting points for constructing and practicing such spatial interventions.
